# A network model of neural activity in essential tremor

**DOI:** 10.1186/1471-2202-16-S1-P7

**Published:** 2015-12-18

**Authors:** Nada Yousif, Michael Mace, Nicola Pavese, Roman Borisyuk, Dipankar Nandi, Peter Bain

**Affiliations:** 1 Division of Brain Sciences, Imperial College London, London, W6 8RF, UK; 2 Department of Bioengineering, Imperial College London, London, SW7 2AF, UK; 3 School of Computing and Mathematics, University of Plymouth, Plymouth, PL4 8AA, UK

## 

**Figure 1:**
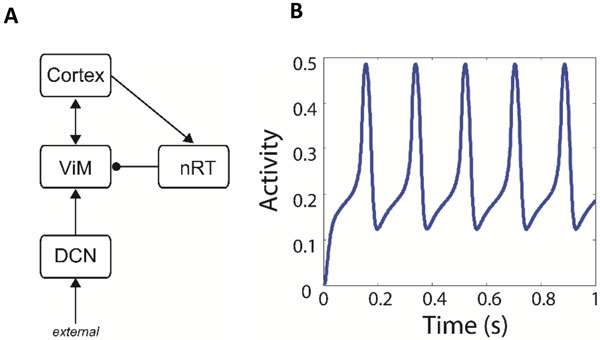
**The structure of the model is shown schematically (A), and consists of the cortex, the ViM nucleus and the reticular nucleus (nRT) of the thalamus, and the deep cerebellar nuclei (DCN)**. The baseline oscillatory activity of the thalamic population is within the ET frequency range (B).

Deep brain stimulation (DBS) is a surgical treatment used for a number of movement disorders, involving the chronic implantation of electrodes into disorder specific regions in the brain. For essential tremor (ET) the targeted brain structure is the ventralis intermedius (ViM) nucleus of the thalamus. ET is a common movement disorder, which affects as many as 4 out of 100 adults over 40 years of age. While the cause of this disorder is unknown, DBS works well, achieving up to 90% improvement in symptoms [1,2]. However, the mechanisms by which the therapeutic effect is obtained are not fully understood, which in turn slows the process of finding the optimal parameter settings which suppress symptoms and minimise unwanted side effects. DBS additionally provides an opportunity to record the pathological neural activity via intraoperative recordings from the implanted electrodes in the form of local field potentials (LFP) whilst simultaneously recording muscle activity from the affected limbs (EMG). Here, we present data which shows peaks in the EMG-LFP cross spectra within the tremor frequency band (4-12 Hz). To understand the effects of a DBS input on such pathological neural activity, we adopt a computational modelling approach using a population representation of the network hypothesised to underlie ET, namely cortex, cerebellum and thalamus (Figure 1 A) and is based on previous descriptions of the essential tremor network [3]. The model is implemented using the Wilson-Cowan approach [4], and was simulated by exploring the parameter space to uncover regions which produced oscillatory thalamic activity and we found that the network exhibited oscillatory behaviour within the tremor frequency range (Figure 1B). By applying an input to the thalamus simulating the effect of DBS (e.g. 150Hz square pulse), we found that these oscillations are suppressed. Therefore, this study shows that the dynamics of the ET network are able to support oscillations at the tremor frequency. Furthermore, the application of a DBS-like input to such a network disrupts synchronous activity, which could explain one mechanism by which DBS achieves therapeutic benefit.
